# Comparison of exenatide with biphasic insulin aspart 30 on glucose variability in type 2 diabetes: study protocol for a randomized controlled trial

**DOI:** 10.1186/s13063-016-1258-8

**Published:** 2016-03-24

**Authors:** Shaoyong Xu, Xiangyang Liu, Jie Ming, Qiuhe Ji

**Affiliations:** Department of Endocrinology, Xijing Hospital, Fourth Military Medical University, 169 Changle Road West, Xi’an, 710032 China

## Abstract

**Background:**

Apart from the mean level of glycemic control, the extent of glucose excursions is another important issue to consider in type 2 diabetes mellitus (T2DM) management. Studies have showed that fluctuations of glucose seem to have more deleterious effects than sustained hyperglycemia in the development of diabetic complications as acute glucose swings activate the oxidative stress. However, until now, no randomized controlled trials have been conducted with the primary aim to evaluate glycemic fluctuation in the comparison between twice-daily exenatide and other treatment paradigms (for example, biphasic insulin aspart 30).

**Methods/design:**

This multicenter, open-label, randomized, parallel trial includes a 1-week screening period and a 16-week treatment period. After the screening period, 150 patients with confirmed type 2 diabetes who are treated with stable, maximum-tolerated doses of metformin will be randomly assigned to one of two groups for antihyperglycemic therapies: exenatide and biphasic insulin aspart 30. The treatment with exenatide will be initiated at a low dose of 5 μg twice a day for 4 weeks and then titrated up to a standard dose of 10 ug twice a day until the completion of the study. The adjustment of insulin dose is instructed to achieve an optimal balance between glycemic control and the risk of hypoglycemia as dictated by clinical practice. The primary outcome is the absolute change of mean amplitude of glycemic excursion from baseline to week 16, which is calculated based on a real-time continuous glucose monitoring system (CGMS).

**Discussion:**

This is the first randomized controlled trial using a CGMS to evaluate glycemic fluctuation between twice-daily exenatide and insulin aspart 30, which will provide beneficial evidence of exenatide usage in patients with T2DM.

**Trial registration number:**

NCT02449603. Date of registration: 11 May 2015.

## Background

Due to the rapid change in the Western diet and sedentary lifestyle, China has become one of the countries with the largest number of people suffering from diabetes in the world. The most recent two nationwide diabetes surveys indicated that the prevalence of type 2 diabetes mellitus (T2DM) has reached 9.7 % and 11.6 % [1, 2], translating into 92.4 million and 113.9 million patients in China [1, 2], respectively.

Uncontrolled T2DM is associated with long-term microvascular complications, such as retinopathy, nephropathy, and neuropathy, as well as cardiovascular events that are dangerous and even fatal. Strict glucose management is thus required. In current clinical practice guidelines, the first-line medication for T2DM is metformin. When oral therapies fail to provide adequate glycemic control, the common treatment paradigm is to initiate insulin therapy [3] such as biphasic insulin aspart 30. For example, nearly 70 % of Chinese patients who are initiating insulin therapy choose premixed insulin [4]. Previous studies have shown that, in combination with metformin, twice-daily biphasic insulin aspart 30 before breakfast and dinner improves glycemic control more than metformin plus glyburide or biphasic insulin aspart 30 alone [5].

An emerging treatment option for patients failing to respond to oral antidiabetic drugs is glucagon-like peptide-1 receptor agonist (GLP-1RA) [3]. Exenatide is an incretin mimetic that shares several glucoregulatory actions with the incretin glucagon-like peptide-1, including the enhancement of glucose-dependent insulin secretion, the suppression of inappropriately elevated postprandial glucagon secretion, the reduction of food intake, and the slowing of gastric emptying [6, 7]. Placebo-controlled studies have shown that exenatide-treated T2DM patients that are suboptimally controlled with oral glucose-lowering agents can exhibit improved glycemic control, together with the additional benefit of sustained weight reduction [8–12].

Apart from the mean level of glycemic control, the extent of glucose excursions is another important issue in T2DM management. Some studies have shown that glucose fluctuations seem to have more deleterious effects than sustained hyperglycemia in the development of diabetic complications as acute glucose swings activate oxidative stress [13, 14]. Recent studies have indicated that glycemic variability might play a role in the pathogenesis of atherosclerosis and may be an independent risk factor for cardiovascular complications in diabetic patients [15]. Previous studies have also suggested that exenatide may provide a better improvement of glucose variability with similar glycemic control than insulin aspart 30 [16]. However, until now, no randomized controlled trial has been conducted with the primary aim to evaluate glycemic fluctuation in the comparison between twice-daily exenatide and other treatment paradigms (for example, biphasic insulin aspart 30).

This randomized controlled study aimed to demonstrate whether exenatide, compared to biphasic insulin aspart 30, could provide a superior improvement of glycemic excursion (assessed by continuing glucose monitor system (CGMS)) and a better improvement of inflammatory and oxidative stress markers after a 16-week treatment in Chinese T2DM patients who failed to improve on metformin monotherapy.

## Methods/design

This multicenter, open-label, randomized, parallel trial, which includes a 1-week screening period and a 16-week treatment period, is planned to start in June 2015 and complete in September 2016.

### Location and setting

Eight study sites will be involved in the multicenter study, including Xijing Hospital, Fourth Military Medical University (Xi’an, China), the First Affiliated Hospital of Xi’an Jiaotong University (Xi’an, China), the Second Affiliated Hospital of Xi’an Jiaotong University (Xi’an, China), Shaanxi Provincial People’s Hospital (Xi’an, China), Chang An Hospital (Xi’an, China), Xi’an Gaoxin Hospital (Xi’an, China), Xi’an Central Hospital (Xi’an, China) and Shaanxi Aerospace Hospital (Xi’an, China).

### Study population and eligibility criteria

A total of 150 subjects will be enrolled. Subjects should fulfil the following criteria:

1. Provide informed consent prior to any study specific procedures.
2. Men and women (nonpregnant and using a medically approved birth-control method) between 18 and 70 years of age at screening.
3. Confirmed type 2 diabetes with history of at least half a year.
4. Treatment with stable, maximum-tolerated doses of metformin (≥1500 mg/d, ≥ 3 months).
5. HbA1c ≥ 7.5 % and ≤ 10.0 % at screening or within 4 weeks prior to screening (by local laboratory).
6. Body mass index: 21 to 35 kg/m^2^.

Subjects should not enter the study if any of the exclusion criteria in Appendix 1 are fulfilled.

### Interventions

The trial will include a 1-week screening period of stable doses of metformin (1500 mg) and a 16-week treatment period. Patient will take the first dose after randomization on Day 1. The investigational products of exenatide and biphasic insulin aspart 30 will be administered through subcutaneous injection. The background product of metformin will be taken orally at 500 mg three time daily until the completion of the study.

Exenatide (AstraZeneca) should be initiated, 60 minutes before breakfast and before dinner, at a low dose of 5 ug twice a day for 4 weeks and then titrated up to a standard dose of 10 ug twice a day until the completion of the study.

Biphasic insulin aspart 30 (Novo Nordisk) will be started at a dose of 0.2 to 0.4 IU/kg, or 10 to 12 IU/d, assigned to before breakfast or before dinner in a 1:1 ratio. The adjustment of the insulin dose will be instructed to achieve an optimal balance between glycemic control and the risk of hypoglycemia as dictated by clinical practice. It will be titrated to glucose targets of fasting plasma glucose (FPG) and predinner plasma glucose < 7 mmol/L. Insulin dose titration will be based on the average self-monitored blood glucose (SMBG) results for 3 days preceding visits, unless hypoglycemia occurs. If hypoglycemia occurs, titration will be temporarily postponed, and the reasons of hypoglycemia will be evaluated. Subjects could not increase the total daily dose by more than 10 U at any time. After visit 2 (week 0), insulin doses will be titrated weekly for the first 12 weeks, then every 2 weeks thereafter according to the titration algorithm (Table 1).

**Table 1:** Prebreakfast and predinner insulin titration regimens

Fasting and/or predinner SMBG PG value (mmol/L)	Adjustment
<5.0	-2 U
5.0 to 7.0	No adjustment
7.1 to 7.7	+2 U
7.8 to 10	+4 U
>10	+6 U

BG, blood glucose; SMGB, self-monitored blood glucose

Glycemic targets for titration: Prebreakfast and predinner PG: 5.0 to 7.0 mmol/L. Prebreakfast dose of biphasic insulin aspart 30 is titrated based on the three mean predinner PG values for the 3 days prior to the visit. Predinner dose of biphasic insulin aspart 30 is titrated based on the three mean fasting PG values for the 3 days prior to the visit. The predinner biphasic insulin aspart 30 titration is not increased if the preceding day’s bedtime SMGB value is 5.6 mmol/L

### Outcomes

The primary and secondary outcomes are shown in Table 2.

**Table 2:** Primary and secondary outcomes

	Outcome measure:
Primary outcome	
To compare the effect of exenatide versus biphasic insulin aspart 30 on glucose variability in T2DM patients inadequately controlled with metformin monotherapy.	Absolute change of MAGE from baseline to Week 16
Secondary outcome	
To compare the effect of exenatide versus biphasic insulin aspart 30 on inflammatory and oxidative stress markers, HbA1c, weight, risk of hypoglycemia and cardiovascular risk markers.	HbA1c at baseline and Week 16
	Hours of hypoglycemia assessing by CGMS at baseline and Week 16
	SMBG at baseline and Week 16
	Blood pressure and lipids at baseline and Week 16
	Body weight, BMI and WC at baseline and Week 16
	Inflammatory markers (MCP-1, hs-CRP) at baseline and Week 16
	Urinary albumin at baseline and Week 16
Safety outcome	
To evaluate the safety and tolerability of exenatide in relation to biphasic insulin aspart 30.	Adverse events/serious adverse events
	Vital signs
	Clinical hypoglycemia
	Collection of clinical chemistry/hematology parameters
	Electrocardiogram

T2DM, type 2 diabetes mellitus; MAGE, mean amplitude of glycemic excursion; CGMS, continuous glucose monitoring system; BMI, body mass index; WC, waist circumference; MCP-1, monocyte chemotactic protein-1; hs-CRP, high-sensitivity C-reactive protein

### Participant timeline

The time schedule for the enrollment, interventions, assessments, and visits for participants are shown in Table 3.

**Table 3:** Study plan detailing the procedures

Study Period	Screening	Treatment							
Visit	V1	V2	V3	V4	V5	V6	V7	V8	V9
		Randomization							
Week	−1	0 (baseline)	1	2	4	8	10	12	16
Day		0	±2	±2	±7	±7	±2	±7	±7
Telephone visit			×	×			×		
Screening/baseline									
Written informed consent	×								
Inclusion/exclusion criteria	×	×							
Demographics	×								
Physical examination	×	×			×	×		×	×
Medical/current conditions	×	×	×	×	×	×	×	×	×
History of diabetes & complications	×								
Lifestyle instruction	×	×	×	×	×	×	×	×	×
Treatment									
Drug dispensing and accountability		×			×	×		×	
Concomitant medications	×	×	×	×	×	×	×	×	×
SMBG			×	×	×	×	×	×	×
Insulin titration			×	×	×	×	×	×	
Safety assessments									
Pregnancy test (urine)	×								×
Physical examination	×	×			×	×		×	×
Vital signs	×	×			×	×		×	×
12-lead ECG	×								×
Hematology panel	×								×
Urinalysis	×								×
Liver function	×								×
Creatinine, BUN, uric acid	×								×
Lipids	×				×				×
Adverse events		×	×	×	×	×	×	×	×
Efficacy assessments									
HbA1c	×								×
Fasting plasma glucose	×	×			×	×		×	×
Fasting insulin and C-peptide		×							×
CGMS		×							×
Standard meal test:2 h PPG, insulin and C-peptide		×							×
BW, BMI and WC	×	×			×	×		×	×
Other assessment									
MCP-1, hs-CRP		×							×
8-Iso-PGF2α		×							×
Urinary albumin		×							×

SMBG, self-monitoring of blood glucose; ECG, electrocardiogram; BUN, blood urea nitrogen; CGMS, continuous glucose monitoring system; PPG, postprandial plasma glucose; BW, body weight; BMI, body mass index; WC, waist circumference; MCP-1, monocyte chemotactic protein-1; hs-CPR, high-sensitivity C-reactive protein; 8-Iso-PGF2α, 8-Iso-prostaglandin F2α

### Sample size estimate

This study is a pilot study, which is planning to recruit 150 patients. This sample size is a justification from statistical considerations as follows:

The hypothesis on the primary endpoint is to test the mean amplitude of glycemic excursion (MAGE) in exenatide group superior to aspart 30 group. According to the literature review and previous studies, the standard deviation (SD) of change of MAGE is conservatively estimated as 3.0 mmol/L (in a pilot study of exenatide, the SD of MAGE is 2.7 to 3.1 mmol/L) [17]. With a two-sided 0.05 alpha level and a 15 % dropout rate, 150 patients with a 1:1 allocation rate (75 patients per group) will offer at least 85 % power to test a minimum 1.6 mmol/L difference on the change of MAGE between the exenatide and aspart 30 groups.

### Assignment of interventions

Patients will be randomized 1:1 to treatment groups via a central randomization system (interactive voice/web response system (IVRS/IWRS)), and the drug will be dispensed accordingly. The study population will be stratified at randomization based on MAGE (<5.6 mmol/l and ≥ 5.6 mmol/l) and the severity of the disease (HbA1c < 9.0 % and ≥ 9.0 %).

Because the study drug (Exenatide) has an obviously different appearance from, and is used differently from, the biphasic insulin aspart 30, a definite blindness is hard and worthless for investigators and patients. Therefore, an open-label study is being conducted. Bias at randomization is reduced by the use of a central randomization system. Moreover, key efficacy parameters (MAGE, HbA1c, FPG, and postprandial plasma glucose (PPG)) will be blinded for the investigator during the course of the study (from randomization to week 16) except for the screening and pre-randomization visit. However, when FPG meets the study drug discontinuation criterion of hyperglycemia, the investigator will be alerted to conduct retest for confirmation.

### Data collection and management

The laboratory parameters that will be measured to assess efficacy and safety at each visit are shown in Table 3. Blood samples for efficacy and safety assessments will be performed on the days of the specified visits in a fasted condition (expect 2-h PPG), prior to administration of study treatment (patients must not take study drug at home on the day of the study visit but must bring it to the study center). Blood sample for 2-h PPG testing will be drawn at 120 min after the first intake of standard meal during the standard meal testing on the days of the specified visits. All blood samples for efficacy assessments in the study will be sent to the central lab for analysis. The blood and urine samples for safety assessments will be performed at local laboratories.

Blood glucose (BG) is monitored in the study sites by a Guardian™ Real-Time CGMS (Medtronic MiniMed Inc., CA, USA), which is used to record glucose levels every 5 min for 72 h consecutively. During the second visit (baseline visit) and the last visit (end of the study), the continuous glucose sensor is inserted subcutaneously in the abdominal periumbilical area, separately. Meanwhile, a blood glucose meter is used to perform sensor calibration four times daily. The participants receive a calibration 2 hours after sensor insertion and then four glucose calibrations at each meal and before bed, and the time of each meal is marked in the consecutive 3-day test. Participants are provided the same mixed meals (30 kcal/kg ideal weight/day, based on dietitian recommendations, including 55 % of calories from carbohydrates, 25 % from fat and 20 % from proteins, with a meal distribution of 1/5, 2/5 and 2/5). They must keep a diary of meals (content and times) and avoid intensive physical activity during both CGMS periods. The dietary pattern using 3-day diet records for each CGMS study period will be checked in all participants. All the parameters of continuous glucose monitoring are calculated from each CGMS output, which is extracted using the CGMS 3.0 software package (Medtronic MiniMed, MMT-7310 version 3.0C (3.0.128), CA, USA). The mean glucose level is calculated as the mean of all the consecutive sensor readings, from which the SD is also calculated. The largest amplitude of glycemic excursion (LAGE) is defined as the maximal sensor glucose level minus the minimal sensor glucose level during each day. The MAGE is used for assessing the intraday glucose variability. MAGE is calculated by measuring the arithmetic mean of the differences in consecutive peaks and nadirs, which are taken into consideration only if they exceed one standard of deviation from the mean [18]. The mean of the daily differences (MODD), calculated as the average absolute difference of paired sensor glucose values during two successive 24-h periods, is used to assess the interday glucose variability.

Instant noodles are used as a standard meal, which includes 85 g of instant noodle equivalent to an energy intake of 500 kilocalories. The procedures for standard meal test are described in Table 4. The fasting plasma glucose will be tested at visits 1 to 6. HbA1c, fasting C-peptide and 2-h PPG will be tested at visit 1, 2 and 6. The baseline is defined as the assessment at Visit 2. The samples are collected after fasting for 12 hours for fasting plasma glucose, fasting C-peptide and HbA1c. Then the participants will be given 85-gram instant noodles and asked to eat up in 8 minutes at visit 2 and visit 6. Two hours later, blood samples will be collected again for plasma glucose.

**Table 4:** Overview of standard meal test assessments

Time	Assessment
-15 ± 5 min	Blood sampling for plasma glucose, insulin, and C-peptide testing
-15 ± 5 min	Administer metformin and exenatide (or biphasic insulin aspart 30)
0 min	Start of ingestion standard meal
+8 ± 2 min	End of ingestion standard meal
+120 ± 10 min	Blood sampling for plasma glucose, insulin, and C-peptide testing

All time points are relative to targeted first mouthful of the standard meal, at Time 0

Physical examination, electrocardiogram, vital signs, and other safety assessments also will be performed using standard methods. Since the two study drugs (exenatide and biphasic insulin aspart 30) are two well-described safe antihyperglycemic medications, and our treatment period is only 16 weeks, some of the safety assessments, like urinalysis, liver function, creatinine, and blood urea nitrogen, are tested only at the baseline and the 16-week safety assessment. Adverse events during all the study period will be collected.

### Statistical methods

The primary analysis population will be based on the full analysis set (FAS) and will include all randomized patients, according to the intention-to-treat (ITT) basis. All supportive analyses will be based on per-protocol set (PPS) analysis set, and the safety endpoints will be analyzed on safety analysis set (SS). The research has no plans to implement interim analysis.

All data will be entered into SPSS 10.0 software (SPSS Inc., Chicago, IL). Analyses by actual therapy include only those patients who are continuing to take their allocated therapy. Data are presented as mean and SD, range, or percentage. Data will be examined for skewness, outliers, and systematic missing data. Transformations will be undertaken as needed. Univariate and multivariate tests for repeated measures (ANOVA) are used to evaluate differences within and between groups over time. Mann–Whitney *U* test is used to evaluate differences between groups, whereas Friedman’s test (variance analysis) followed by Wilcoxon paired test is used to evaluate differences within groups. *P* < 0.05 is considered significant.

All efficacy endpoints will be analyzed in FAS, using a covariance model. Point estimates and SD for the mean change within each treatment group will be estimated. Difference within and between groups over time will be analyzed using covariance test. A last observation carried forward (LOCF) analysis will be presented for each efficacy assessment as sensitivity analysis unless otherwise specified.

The safety endpoints will be analyzed on SS. Adverse events will be summarized. The incidence of adverse events will be summarized by body system organ class and preferred term. Analyses for safety and tolerability endpoints will be summarized using descriptive statistics for continuous variables or frequency counts and percentages for categorical variables.

### Ethics and dissemination

The trial was approved by the Independent Ethics Committee, First Affiliated Hospital of Fourth Military Medical University (ID: KY20150319, KY20150505-1) and was registered (registration date: 11 May 2015) in the United States National Institutes of Health Clinical Trials Registry (ClinicalTrials.gov, identifier: NCT02449603). The study will be supervised by Excellence Future International Consulting Co., Ltd. The findings of this trial will be submitted to a peer-reviewed journal. Abstracts will be submitted to relevant national and international conferences.

Each subject will provide signed and dated informed consent before conducting any procedure specifically for the study. Participation in this study is strictly confidential. Any information that is published will not reveal the identity of the subjects.

## Discussion

An unmet need exists for diabetes research with interest in glucose variability assessed by CGMS, as glucose variability is suggested to lead to the development of long-term complications [19] and MAGE assessed by CGMS is the “gold standard” to evaluate glucose variability.

In American Diabetes Association (ADA), European Association for the Study of Diabetes (EASD), and Chinese Diabetes Society (CDS) guidelines, both insulin and GLP-1 RA could be the initial option when oral therapies fail to provide adequate glycemic control, and nearly 70 % Chinese patients choose premixed insulin as the initial insulin therapy [4].

Previous studied have demonstrated that, compared with insulin aspart 30, exenatide showed a better effect on glycemic fluctuation assessed by seven-point SMBG with similar glycemic control and lower risk of hypoglycemic [16, 20]. For example, in a 52-week, open-label, non-inferiority trial, patients failing to reach treatment goals with metformin and sulphonylurea were randomized to exenatide (n = 253; 5 μg twice daily (BID) 4 weeks, 10 μg thereafter) or premixed insulin (n = 248; BID doses titrated for optimal glucose control). The results demonstrated that exenatide BID has a similar glycemic effect to insulin aspart 30 assessed with HbA1c in T2DM patients. More importantly, the results demonstrated that exenatide-treated patients had significantly greater reductions in postprandial glucose excursions (the difference between preprandial and postprandial plasma glucose levels) following the morning (*P* < 0.001), midday (*P* < 0.002), and evening meals (*P* < 0.001) than patients treated with premixed insulin assessed with seven-point SMBG [16]. However, in these studies, MAGE was not the primary endpoint, or CGMS was not used.

The statistically significant and clinically relevant reduction on glucose variability with exenatide was associated with the profile of safety and tolerability superior to biphasic insulin aspart 30. Data establishing the clinical efficacy of exenatide are based on two core Phase 3 studies: one glimepiride-controlled pilot study with glycemic variability assessed by CGMS, which only included 12 patients [17], and one biphasic insulin aspart 30-controlled study, where glycemic excursion was assessed by SMBG [16]. The key results of these studies were that treatment with exenatide resulted in consistent, clinically meaningful, and statistically significant greater reductions in glucose excursions, in addition to being associated with non-inferior glycemic control relative to the active comparator.

This is the first randomized controlled trial using CGMS to evaluate glycemic fluctuation between twice-daily exenatide and insulin aspart 30, which will provide beneficial evidence to exenatide usage in patients with T2DM.

### Trial status

This trial is currently ongoing and actively recruiting patients. The first participant was enrolled on 10 December 2015.

## Appendix 1

### Exclusion criteria

1. Women who are pregnant, intending to become pregnant during the study period, currently lactating females, or women of child-bearing potential not using highly effective, medically approved birth control methods.
2. Diagnosis or history of a) type 1 diabetes mellitus, diabetes resulting from pancreatic injury, or secondary forms of diabetes, for example, acromegaly or Cushing’s syndrome. b. Acute metabolic diabetic complications such as ketoacidosis or hyperosmolar coma within the past 6 months.
3. Previous treatment with any dipeptide peptidase-4 (DPP4) inhibitor or GLP-1 receptor agonists within the past year.
4. History of hypersensitivity reaction (for example, anaphylaxis, angioedema, or exfoliative skin conditions) to dipeptide peptidase-4 inhibitor (DPP4) or acarbose.
5. Treatment with any antidiabetic medication for more than 7 consecutive days other than metformin in the 3 months prior to screening.
6. Treatment with systemic glucocorticoids (oral or intravenous) for more than 7 consecutive days within the past 6 months.
7. Triglycerides (fasting) > 4.5 mmol/L (>400 mg/dL) at screening or within 4 weeks prior to screening (by local laboratory).
8. Patients with clinically apparent liver disease characterized by either of the following: a) ALT or AST > 3x upper limit of normal (ULN) confirmed on two consecutive measurements (by local laboratory) within 4 weeks prior to screening period; b) impaired excretory (for example, hyperbilirubinemia) and/or synthetic function, or other conditions of decompensated liver disease such as coagulopathy, hepatic encephalopathy, hypoalbuminemia, ascites and bleeding from esophageal varices; or c) acute viral or active autoimmune, alcoholic, or other types of hepatitis.
9. Patients with moderate/severe renal impairment or end-stage renal disease (estimated glomerular filtration rate ≤ 60 mL/min calculated by using the abbreviated equation developed by the Modification of Diet in Renal Disease (MDRD) study with modification for the Chinese population) at screening or within 4 weeks prior to screening (by local laboratory).
10. Congestive heart failure defined as New York Heart Association (NYHA) class III or IV.
11. Significant cardiovascular history within the 3 months prior to screening, defined as myocardial infarction, coronary angioplasty or bypass graft(s), valvular disease or repair, unstable angina pectoris, transient ischemic attack, or cerebrovascular accident.
12. History of chronic pancreatitis or idiopathic acute pancreatitis.
13. History of gastrointestinal disease, including gastroenterostomy, enterectomy, Roemheld Syndrome, severe hernia, intestinal obstruction, or intestinal ulcer.
14. History of genetic galactose intolerance, Lapp lactase deficiency, and glucose-galactose malabsorption.
15. History of medullary thyroid carcinoma.
16. Diagnosed and/or treated malignancy (except for basal cell skin cancer, in situ carcinoma of the cervix, or in situ prostate cancer) within the past 5 years.
17. History of organ transplant or acquired immunodeficiency syndrome (AIDS).
18. History of alcohol abuse or illegal drug abuse within the past 12 months.
19. Potentially unreliable patients and those judged by the Investigator to be unsuitable for the study.

## Abbreviations

ADA: American Diabetes Association
BG: blood glucose
BID: twice daily
BMI: body mass index
BUN: blood urea nitrogen
BW: body weight
CDS: Chinese Diabetes Society
CGMS: continuous glucose monitoring system
EASD: European Association for the Study of Diabetes
ECG: electrocardiogram
FAS: full analysis set
FPG: fasting plasma glucose
GLP-1RA: glucagon-like peptide-1 receptor agonist
hs-CPR: high-sensitivity C-reactive protein
IVRS/IWRS: interactive voice/web response system
LAGE: largest amplitude of glycemic excursion
LOCF: last observation carried forward
MAGE: mean amplitude of glycemic excursion
MCP-1: monocyte chemotactic protein-1
MODD: mean of the daily differences
PPG: postprandial plasma glucose
PPS: per-protocol set
SD: standard deviation
SMBG: self-monitored blood glucose
SS: safety analysis set
T2DM: type 2 diabetes mellitus
WC: waist circumference
8-Iso-PGF2α: 8-Iso-prostaglandin F2α
